# The Multifaceted Roles of Plant Hormone Salicylic Acid in Endoplasmic Reticulum Stress and Unfolded Protein Response

**DOI:** 10.3390/ijms20235842

**Published:** 2019-11-21

**Authors:** Péter Poór, Zalán Czékus, Irma Tari, Attila Ördög

**Affiliations:** Department of Plant Biology, University of Szeged, Közép fasor 52, H-6726 Szeged, Hungary; czekus.z@bio.u-szeged.hu (Z.C.); tari@bio.u-szeged.hu (I.T.); aordog@bio.u-szeged.hu (A.Ö.)

**Keywords:** binding protein, cell death, pathogenesis-related genes, systemic acquired resistance, tunicamycin

## Abstract

Different abiotic and biotic stresses lead to the accumulation of unfolded and misfolded proteins in the endoplasmic reticulum (ER), resulting in ER stress. In response to ER stress, cells activate various cytoprotective responses, enhancing chaperon synthesis, protein folding capacity, and degradation of misfolded proteins. These responses of plants are called the unfolded protein response (UPR). ER stress signaling and UPR can be regulated by salicylic acid (SA), but the mode of its action is not known in full detail. In this review, the current knowledge on the multifaceted role of SA in ER stress and UPR is summarized in model plants and crops to gain a better understanding of SA-regulated processes at the physiological, biochemical, and molecular levels.

## 1. Introduction

### 1.1. ER Stress

The eukaryotic endoplasmic reticulum (ER) has multiple cellular functions, such as protein synthesis, assembly, folding, and export. The lumen of the ER is a specific environment, which contains a high concentration of Ca^2+^, playing a role in various cell signaling events. In addition, the lumen of the ER is also an oxidative environment, which regulates the formation of disulphide bonds and proper folding of proteins. Finally, the newly synthesized and correctly folded proteins are loaded for transfer from the ER into the cytosol [1,2]. To maintain the balance between protein folding and transport and the capacity of ER, many Ca^2+^-dependent molecular chaperones cooperate in the ER, such as calreticulin (CRT) and calnexin (CNX). The binding protein (BiP; glucose-regulated protein 78, Grp78), Grp94, protein disulfide isomerase (PDI), and peptidyl propyl isomerase (PPI) are also central players in protein folding quality control [2,3,4,5,6]. Several abiotic (e.g., high light, high temperature, drought, salt, osmotic and heavy metal stress) and biotic stresses (e.g., bacterial and fungal pathogens, viruses) can induce ER stress in plants [7,8,9,10]. Namely, disturbances in ER homeostasis under stress conditions, including those of cellular redox regulation, cause ER stress by the accumulation of unfolded and misfolded proteins that triggers an evolutionarily conserved response, termed the unfolded protein response (UPR). UPR is a protective response to maintain the cellular homeostasis by regulating the expression of a variety of genes (e.g., chaperones) and by reducing protein loading to the ER and enhancing ER-associated protein degradation (ERAD). These processes improve the protein folding capacity and remove the unfolded or misfolded proteins from the ER [10,11,12,13]. Programmed cell death (PCD) and autophagy are also associated with ERAD response under prolonged and chronic stress effects [14,15,16,17]. Recently, it has been suggested that the plant hormone salicylic acid (SA) induces UPR in plants, but the underlying mechanisms are not completely known yet [10]. To test the potential role of SA in UPR, exogenous application of several chemicals like tunicamycin (Tm, the inhibitor of N-glycosylation of secreted glycoproteins), dithiothreitol (DTT, the inhibitor of the formation of disulphide bonds during protein folding), and azetidine-2-carboxylic acid (AZC, a proline analogue that can interfere with the formation of native protein structure) have been used under laboratory conditions [18]. At the same time, it has been found that after the treatment with Tm caused a four-fold increase in the SA content of *Arabidopsis* [19].

In this review, the current knowledge on the multifaceted role of SA in ER stress and unfolded protein responses will be summarized in model plants and crops to gain a better understanding of SA-regulated processes at the physiological, biochemical, and molecular levels. This knowledge can add a new aspect to the understanding of plant ER stress and UPR signaling and its crosstalk with plant immune responses.

### 1.2. Basic Properties of UPR

**Figure 1:**
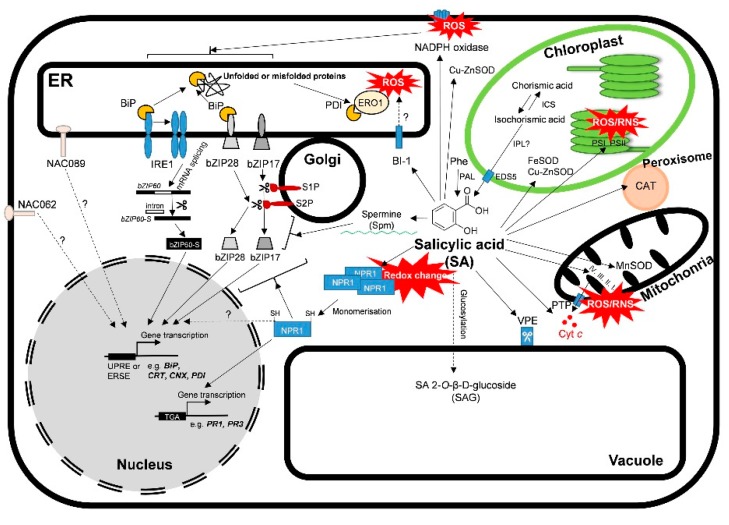
A schematic illustration of unfolded protein response (UPR) and the effects of salicylic acid (SA) under stress condition in plants. The accumulation of unfolded proteins in the ER leads to the conformational changes and activated RNAse function of IRE1 (inositol-requiring enzyme 1), which mediates an unconventional splicing bZIP60 transcription factor mRNA. Spliced bZIP60 mRNA is translated to an active transcription factor and translocated to the nucleus and upregulates *UPR* genes containing unfolded protein response element (UPRE) and ER stress element (ERSE) in their promoters. bZIP28 and bZIP17 are activated by Golgi proteases (S2P and S1P). Then, the cleaved forms of bZIPs translocate into the nucleus and binds to ERSE to activate the *UPR* gene expression. NAC062 and NAC089 also undergo proteolytic cleavage and translocate to the nucleus to promote the transcription of *UPR* or cell death genes. SA has multifaceted roles in the regulation of defense or cell death processes in plants. SA is synthesized by phenylalanine ammonia-lyase (PAL) from L-phenylalanine (Phe) or in the isochorismate (IC) pathway by isochorismate synthase (ICS). Then through the activity of isochorismate pyruvate lyase (IPL) in the chloroplast, it is translocated to the cytosol by EDS5. SA induces high production of reactive oxygen (ROS) and nitrogen species (RNS) in chloroplast and mitochondria and activates NADPH oxidase, respectively. Simultaneously, SA activates various antioxidant enzymes, such as superoxide dismutases (SOD). SA induces cytochrome *c* (Cyt *c*) release from the mitochondrial inner membrane by the permeability transition pore (PTP), decreases the transcript levels of Bax inhibitor-1 (BI-1), and increases the expression of vacuolar processing enzymes (VPEs) inducing cell death. Moreover, SA has a significant effect on polyamine levels (e.g. that of spermine), influencing ER stress in plants. SA changes the redox homeostasis and induces the reduction and monomerization of NPR1, which is translocated to the nucleus where it binds to specific TGA transcriptions factors, inducing the expression of SA-induced defensive response genes (PRs). SA can be inactivated and stored as SA O-β-glucoside (SAG) in the vacuole. *Detailed description and references are in the text*.

The cytoprotective UPR is initiated by ER-resident stress sensors located in the ER membrane (Figure 1). One of them, the inositol-requiring enzyme 1 (IRE1)-mediated unconventional splicing of basic leucine zipper (bZIP) 60, is the most conserved in eukaryotes [18,20,21]. However, the activation mechanism of plant IRE1 has not been shown in full detail. It was well demonstrated in yeast and animals that the sensor domain of IRE1 binds to the ER-luminal BiP while the full-length bZIP60 is anchored in the ER membrane under normal conditions. The accumulation of unfolded proteins leads to BiP dissociation from IRE1. The released IRE1 is firstly dimerized and then oligomerized after the binding by its luminal domain to the hydrophobic domain of the unfolded proteins. In *Arabidopsis*, two isoforms of IRE1, *IRE1a* and *IRE1b*, are found. It was observed that IRE1b but not IRE1a expressed heterologously in yeast cells showed the oligomerization structure and clustering, indicating the possible conserved step of IRE1 activation in plants, respectively [22,23,24,25]. The activated RNAse function of IRE1 results in the splicing of bZIP60 mRNA and bulk degradation of selected mRNAs through regulated IRE1-dependent decay (RIDD) in animals, yeast, and plants. Spliced bZIP60 mRNA is translated to an active transcription factor (TF) and the active bZIP60 protein is translocated to the nucleus and upregulates *UPR* genes containing unfolded protein response element (UPRE) and ER stress element (ERSE) in their promoters [10,20,21,22,23,24,25]. 

The activation of ER membrane-anchored TF bZIP28 and the plant B-cell lymphoma2 (Bcl-2)-associated athanogene 7 (BAG7) protein is another mode to control ER stress in plants. Both proteins are anchored to the ER membrane by interactions with BiP under unstressed conditions. Like IRE1, bZIP28 is also activated through the stress-induced accumulation of unfolded proteins in the ER lumen. In response to ER stress, BiP dissociates from bZIP28 and the released bZIP28 translocates from ER to the Golgi through the coat protein II (COPII) vesicles, where it is proteolytically cleaved by site-2TF protease (S2P) but not by site-1 protease (S1P). The cleaved form of bZIP28 translocates into the nucleus and binds to ERSE to activate the *UPR* gene expression [26,27,28,29,30]. BAG7 is also released from the ER membrane by an unknown protease, then it is sumoylated and enters the nucleus, where it interacts with WRKY29 transcription factor and regulates the expression of various chaperone proteins to mitigate ER stress [31]. Another ER membrane-associated transcription factor is *bZIP17*, which is closely related to *bZIP28*. It was found that *bZIP17* could be activated by salt stress in *Arabidopsis* in a manner similar to *bZIP28*. Basically, AtbZIP17 is inserted into the ER lumen. Under stress condition, it is transported firstly to the Golgi apparatus where it is cleaved by the Golgi-localized AtS1P protease, and the N-terminus of AtbZIP17 enters the nucleus to activate stress-responsive genes [12,21].

Another TF, the plant-specific NACs (no apical meristem (NAM), *Arabidopsis* transcription activation factor (ATAF), cup-shaped cotyledon (CUC)) have recently been identified as an important regulators of ER stress responses [8,32,33]. In total, 117 *NAC* genes have been found in the *Arabidopsis* genome, which participate in several developmental and stress-induced processes [10,34]. NAC062 (localized to the plasma membrane) and NAC089 (localized to the ER membrane) undergo proteolytic cleavage under ER stress and translocate to the nucleus to promote the transcription of *UPR* or *PCD* genes. NAC089 is dependent on both IRE1/bZIP60 and bZIP28 pathways and plays a role in PCD [32,33]. NAC062 and NAC103 are also controlled by IRE1/bZIP60, inducing the expression of defense genes under stress conditions [32,35].

Finally, protein kinase RNA-like ER kinase (PERK)-mediated translational inhibition was well characterized in mammals, but no PERK homologues have been identified in plant genomes until now [8,10].

## 2. SA as an ER Stress Signaling Regulator in Plants

The phenolic compound salicylic acid (SA) plays a crucial role in plant defense signaling upon various abiotic and biotic stressors [36,37]. It is required for the establishment of both local and systemic acquired resistance (SAR) after pathogen attack. The elevated concentration of SA under stress conditions induces the accumulation of reactive oxygen species (ROS), leading to oxidized proteins and cell death in the infected tissues [38]. Besides, SA induces expression and accumulation of pathogenesis-related (PR) proteins, which requires optimal coordination and regulation of protein secretory machinery to ensure folding, modification, and transport of PR proteins [39,40]. Thus, SA plays a dominant role in ER stress signaling and regulating UPR under stress conditions [41], but the mode of its action is not known in full detail. In addition, there are contrasting findings from different experiments in the case of SA-mediated UPR. It has to be mentioned, however, that the experimental setups cannot be excluded because it is well known that the action of SA is highly dependent, e.g., on its applied or internal concentrations, on the duration and the mode of the application, on the investigated plant species and organs as well as on the light intensity and daytime of SA treatment [42]. Furthermore, the crosstalk between SA and other plant hormones (e.g., ethylene and jasmonic acid) can overwrite the outcome of defense signals and plays a role in the regulation of UPR [43,44]. From this aspect, a physiological approach is also necessary to draw a more complex picture of the role of SA in ER stress and UPR. In this section, the SA-mediated ER stress signaling is summarized to understand the multifaceted role of SA in this process.

Jelitto-Van Dooren et al. [39] postulated firstly the relationship between ER stress and SA-mediated defense responses and described a spatiotemporal change. They observed that plant cell wall-degrading enzymes (CDEs) secreted by the bacterial pathogen *Erwinia carotovora* induced the expression of the β-1,3-glucanase (*PR3*) gene 4 h after incubation and reached a maximum after 8 h. Nevertheless, *BiP*, *PDI*, and *CRT* transcripts accumulated more rapidly, reaching a maximum after 2 h of CDE incubation both in locally treated tobacco leaves as well as in untreated/systemic distal leaves with the same timing and intensity. However, this CDE-induced *BiP* expression was not dependent on SA based on the use of an SA-insensitive mutant of *Arabidopsis* (*sai1*) and could be regulated by other phytohormones, such as ethylene or jasmonic acid. The authors concluded that *BiP* gene expression during plant–pathogen interactions is required to allow efficient PR protein synthesis because more ER chaperones are required for the synthesis, folding, and transport of defense-related proteins [39]. Later, Wang et al. [40] found that the SA-induced various components of ER stress and UPR during the development of SAR are regulated by the SA-induced master regulator protein NPR1 (nonexpressor of pathogenesis-related (*PR*) genes 1) in *Arabidopsis*. Based on microarray experiments, genes of the *Sec61* translocon complex, which provides a channel for proteins to cross the ER membrane, and a signal recognition particle (SRP) receptor were upregulated. In addition, chaperones, such as *BiP2*, *GRP94*, as well as co-chaperones, including defender against apoptotic death 1 (*DAD1*), *CNXs*, *CRTs*, and *PDIs*, were upregulated in an NPR1-dependent manner. The authors suggested that SA primes the ER capacity to assist in the production, folding, and transport of defense proteins, such as PR1. Consistent with this hypothesis, the expression of *BiP2* was induced before the accumulation of *PR1* [40]. It is well known that SA induces the reduction and monomerization of NPR1, which is translocated to the nucleus and induces the expression of *PRs* through interaction with the TGA TFs at the promoters of *PR* genes [43,45]. However, in other experiments, Wang et al. [40] observed that genes encoding ER-resident proteins are not upregulated by TGA TFs but by TL1-binding transcription factor 1 (TBF1). TBF1, in response to infections, plays a role in the rapid reprogramming of transcription from growth to defense responses [46]. The TGA family of bZIP TFs takes part in the regulation of these defense responses of plants. Unfortunately, only the function of clade I TGA factors, which are independent of NPR1 [47], were investigated upon ER stress [48]. The potential role of TGA clade II and III will be elucidated in the future. At the same time, *tga1-1 tga4-1* mutant seedlings showed increased sensitivity to Tm, which was associated with the upregulation of ER-resident genes encoding *BiP1/2* and *BiP3* chaperones, suggesting that the loss of clade I TGA factors does not impair the IRE1/bZIP60 branch of UPR signaling but impairs ER-based protein folding and/or secretion in an NPR1-independent manner [48].

It has also been found that SA (0.5 mM) and Tm (5 mg mL^−1^) induced not only *BiP2* but also *BiP3* transcript levels, but Tm induced the expression of both selected chaperon coding sequences more significantly compared to SA in *Arabidopsis* [49]. Interestingly, SA did not induce *BiP3* expression in a *bZIP60* knockout mutant or in an *ire1a ire1b* double mutant, and the transcript levels of *PR1* and *BiP2* also did not change after SA treatment. In addition, *bZIP60s* and *BiP3* were not induced in *NahG*, an SA-deficient transgenic plants. These observations confirmed that SA induced the activation of the IRE1–bZIP60 pathway and thus *BiP3* expression [49]. Surprisingly, it has also been demonstrated that bZIP60-dependent induction of *UPR* genes (*BiP2* and *BiP3*) by SA is independent of NPR1 by the use of *npr1-1* mutants, where levels of *PR1* transcripts did not increase after 5- or 10-h-long SA treatments [49]. Furthermore, it has also been revealed that SA activates not only *bZIP60* but also *bZIP28* independently of NPR1 after 2 h, but bZIP28 levels decreased after 10 h, suggesting that bZIP28 is activated earlier than bZIP60 under these experimental conditions [49]. There were no differences in the induction profiles of *BiP2*, *BiP3*, and *CNX1* in a T-DNA insertion mutant, *hsfb1-1*, suggesting HsfB1-independent (the major molecular switch for the plant growth-to-defense transition) regulation of UPR by SA under these experimental conditions [49]. It has to be noted that the daytime of SA application and light intensity is not known in these works, but it is well known that the effect of SA depends on these external and internal factors [50,51]. Despite this finding, the spliced form of bZIP60 has been observed at 30 min and the maximum after 2 h upon 0.5 mM SA treatment, but it decreased after 5 h of SA application in 7-day-old *Arabidopsis* seedlings [52]. This change in bZIP60 activation suggests that it is a dynamic process because it has been found that the wash-out of SA led to a complete loss of the spliced form of bZIP60 and the re-addition of SA led to an increase of the spliced bZIP60 form again [52]. Surprisingly, the result of Parra-Rojas et al. [52] suggests that the effects of SA on the splicing of bZIP60 is somehow linked to the function of bZIP17 because the level of spliced bZIP60 was higher in *bZIP17* mutants.

Recently, it has been confirmed that Tm-induced ER stress is regulated by NPR1 because the transcriptional role of bZIP28 and bZIP60 in ER stress responses is antagonized by NPR1 [53]. Moreover, the authors suggested that this action could be independent of the role of NPR1 in SA-mediated defense, because *npr1* mutants displayed enhanced resistance to chronic ER stress in the root growth of *Arabidopsis* and Tm treatment did not cause the accumulation of a free and conjugated form of SA. Furthermore, the transcript levels of TBF1-dependent SA-induced genes (*TGA3*, *PAD4*, and *CRT3*) did not change in Col-0 plants, but the transcript levels of *CNX1*, *BiP2*, and *PDI* showed enhanced induction in *npr1* mutants compared with the wild-type plants [53]. Moreover, the authors also demonstrated that Tm-induced ER stress caused a more negative redox potential of the cytosol similar to earlier observations in the case of SA treatment (0.5 mM) and induced the translocation of NPR1 from the cytosol to the nucleus, where NPR1 interacts with bZIP28 and bZIP60 and suppresses the transcriptional activity of these TFs during UPR [53]. Changes in the redox state of cells under stress conditions could be a significant cellular event. Basically, SA accumulation alters the redox potential in the cytosol, resulting in a conformational change of NPR1 from an oligomeric form to a monomeric form and thus causing nuclear translocation and therefore the reprogramming of transcription [45]. Changes in the redox status of cells upon Tm could be interesting because Tm eliminates the N-glycan present in glycoproteins and significantly affects the folding assisted by ER quality control. At the same time, DTT, similar to SA, alters also the redox balance of the cell [18]. However, accumulation of ROS leading to oxidized proteins can also induce UPR after the Tm treatment [13]. ROS generation by ER luminal oxidoreductase 1 (ERO1), the mitochondria-, and/or plastid-originated ROS [54] and NADPH-oxidase activity-dependent ROS [55] suggest a potential link between ER and other organelles in the oxidative processes. In this relation, SA could be an important signaling compound because SA has a significant effect on ROS production in a time- and concentration-dependent manner in these cell compartments [56,57,58]. However, the direct effects of SA on ERO1 and the relationship between ER and other organelles, which generate ROS, is not known. 

The role of bZIP28 and bZIP60 has also been confirmed in SA-mediated ER stress signaling with the interaction of CPR5 (constitutive expresser of pathogenesis-related genes-5), a plant-specific master regulator of growth and defense, which represses the accumulation of SA [59]. In the case of elevated SA in *cpr5* mutants, the IRE1–bZIP60 arm of ER stress is required for the growth inhibition of *Arabidopsis* seedlings. The expression of *BiP3* was also enhanced in *cpr5*, but it was significantly reduced in a *cpr5 bzip28 bzip60* triple mutant [59]. Moreover, it has also been shown that CPR5 plays a role in the UPR induced by Tm treatment after 12 days. However, CPR5 is a negative modulator of the UPR by modulating the bZIP60/bZIP28 arms of ER stress dependently on endogenous SA under stress conditions. In addition, it has also been demonstrated that there is a physical interaction between bZIP60, bZIP28, and CPR5 at the protein level. It can be concluded that CPR5 is a positive modulator of growth under normal conditions, but it acts by antagonizing SA-dependent growth inhibition through UPR modulation under stress condition [59]. 

There is a strong connection between other ER stress signaling elements and SA. The *Arabidopsis* genome encodes two *IRE1s* (*IRE1a* and *IRE1b*) with different physiological roles. Moreno et al. [60] observed that 4-h-long SA treatment (0.5 mM) induced the expression of both *IRE1a* and *IRE1b* genes. The use of several *ire1a* and *ire1b* mutant and transgenic plants demonstrated that IRE1a plays a predominant role in the secretion of PR proteins upon SA treatment. Mutants of *ire1a* showed enhanced susceptibility to *Pseudomonas syringae* pv. *maculicola* and these plants were not able to establish SAR, whereas *ire1b* mutants were unaffected in these responses. At the same time, IRE1b played a major role in a bZIP60 processing event after Tm treatments. The authors demonstrated that SA-dependent induction of *BiP1/2*, *CRT2*, and *UTr1* was abolished in plants lacking both members of functional IRE1, but the expression of *BiP1/2*, *UTr1*, as well as *PR1* did not change after 3 h in *bzip60* mutants, suggesting bZIP60-independent functions in plant immunity and the potential role of other TFs in this process [60].

Mechanisms of the defense responses can be different in *Arabidopsis* and in another plant species, such as rice (*Oryza sativa* L.). Firstly, 0.1 mM SA-induced activation of OsbZIP74 (also known as OsbZIP50)—an important ER stress regulator in a monocot plant, rice—was observed within 1 h in root cells [61]. In contrast to *Arabidopsis*, IRE1 mediates unconventional splicing of OsbZIP50 in rice, thus inducing ER stress-related factors, such as the ER chaperone *BiP* and counterparts of ER stress signaling, *OsbZIP39* and *OsbZIP60* [62]. At the same time, the endogenous level of SA is much higher in rice than in *Arabidopsis* [63], suggesting the potential concentration-dependent role of SA in ER stress response. OsWRKY45, which is absent from *Arabidopsis*, is an SA-regulated TF and plays a role in the activation of defense response genes upon pathogen infection [64]. Treatment with Tm induced the expression of *OsWRKY45* after 4 h in rice, which was suppressed by chemical chaperon 4-phenylbutyric acid (4-PBA). This induction of *OsWRKY45* was OsbZIP50 dependent upon Tm treatment but it did not depend on OsbZIP50 in the case of application of 0.5 mM SA. Interestingly, co-treatment with Tm and SA was additive to the expression of *OsWRKY45* and *PR1a*, but the transcript levels of *OsBiP1-5* and *OsHSP70* were suppressed by the addition of SA to Tm-treated rice plants. Based on these results, it has been concluded that OsWRKY45 induces the expression of these target genes, which is the priming effect before the activation of SA-activated defense responses. Moreover, it has also been found that ER stress induced by DTT and Tm downregulates the expression of some *PR* genes in an OsIRE1-dependent manner, which can be a protective mechanism by lowering the secretory burden on the ER under stress conditions [65]. Simultaneously, exogenous application of 0.5 mM SA can overwrite the Tm-induced UPR in *Arabidopsis thaliana*. Co-treatment with SA and Tm or DTT significantly decreased transcription levels of *AtBiP3* and *AtbZIP60* after 3 h in root tissues similarly to 4-PBA treatments. These results confirmed that this UPR-suppressive effect of this concentration of SA can be conserved between rice (a monocot) and *Arabidopsis* (a dicot) plants [66]. However, an investigation of the concentration- or time-dependent effects of SA could provide further data to understand the relationship between ER stress and SA-induced defense responses in crops. Interestingly, Tm + SA treatment similarly decreased the expression of *BiP3* after 2 days in roots but not in leaves based on histochemical gene expression analysis of *Arabidopsis* seedlings [66]. These results suggest the potential organ-dependent effects of SA in the regulation of ER stress and UPR, which could be analyzed in the future. An investigation of the changes in different organs and the potential interaction between organs could be important research aims because organ-dependent changes in the level of splicing of bZIP60 have been observed earlier upon heat stress in *Arabidopsis* [52].

Proteolytic activation of a plasma membrane-tethered NAC (NAM/ATAF1/2/CUC2) TF NTL6 is induced by cold stress but not by exogenously applied SA (0.1 mM) in *Arabidopsis*. NTL6 can directly bind to a conserved sequence in the promoters of cold-responsive *PR* genes and induce the expression of *PR1*, *PR2*, and *PR5* under cold stress independently of NPR1/TGA-mediated SA signaling [67]. An analysis of the role of various NAC TFs in SA-dependent and induced defense will provide new research topics in the future.

## 3. SA-Regulated Chaperons: Survival or Death

SA plays an important role in relaying the pathogen signal to activate defense reactions, such as the synthesis of PR proteins and accumulation of ROS, in the development of hypersensitive reaction (HR) or SAR [38]. Since SA is an important signaling molecule in these defense reactions of plants, its effect on UPR is a major topic in plant science. UPR is dependent on molecular chaperones, which are the key components responsible for protein folding, assembly, translocation, and degradation under normal and stress conditions [68]. However, BiPs have diverse functions; among them, the best-known function is their molecular chaperone activity, but they have a central role in ER stress and UPR, which is essential in plant developmental and immunity processes [6]. At the same time, several findings suggest that BiP induction was independent of *PR* gene induction and SA at the early stage of plant–pathogen interaction, because chaperons are required to support PR protein synthesis in the later phase of the infection [39]. Other authors observed that SA plays a dominant role in the induction of several chaperone-coding genes, such as *BiP2* and *BiP3* in *Arabidopsis* [49] or in the upregulation of *BiP*, *CNX*, and *PDI* in soybean plants [69]. The extremely high concentration of SA (5 mM) also induced the expression of both *BiP* and *PDI* in tobacco leaves [70]. It can be concluded that the selected and applied concentration of SA or the internal concentration of SA in the different plant species (e.g., in rice) [71] could determine the outcome of the stress responses of plants and result in different scientific results. The high concentration of SA induces cell death in plants (e.g., at 1 mM in tomato), but simultaneously, defense responses can also be activated [72]. Thus, the protective mechanisms are dependent on the strength and duration of the stress. Based on these observations, mild and prolonged chronic ER stress have been distinguished [14]. Prolonged and/or chronic ER stress is associated with the generation of ROS and cell death-promoting Ca^2+^ signaling, but the potential relationships with other organelles (e.g., mitochondria, chloroplast, and vacuole) still require more in-depth studies [14]. Investigation of these organelles upon SA could be crucial to understand the role of SA in ER stress and UPR [73]. Thus, the concentration- and time-dependent effects of SA could be essential to survive or to induce cell death. In the case of biotic stress, SA accumulation and high levels of PR1 and BiP proteins have been reported many days after *Pseudomonas syringae* infection during SAR development [74]. In contrast, cell death-inducing concentration of DTT increased the transcript levels of *BiP*s, *GRP94*, *CNX*, and *PDI*s genes but decreased the expression of *PR* genes in wheat seedlings 2 days after treatment [75]. It is also very important that the expression of *PDI* and *BiP* genes is highly dependent on plant tissues under untreated conditions [70], which can also be determined by SA-mediated signaling. However, the dual function of BiP in modulating development and HR has also been reported in soybean and tobacco plants [69]. In soybean transgenic lines (35S::BIP4 and 35S::BiP2), the overexpression of functional BiP and downregulation of the antioxidant system, protein degradation, and cell death-associated genes but upregulation of defense and immune system-related genes, such as *PR* and lignin biosynthetic process genes, can be seen. Interestingly, these lines contained more SA compared to wild-type plants. *BiP*-overexpressing lines displayed delayed leaf senescence under normal conditions based on changes in photosynthetic pigment concentrations. During senescence, UPR was activated, but the expression of *BiP*, *CNX*, *PDI*, and *IRE1* homologs were lower in BiP-overexpressing lines compared to the wild type, suggesting a feedback mechanism that involves the monitoring of BiP protein levels. Although *BiP* overexpression downregulated cell death-associated genes, inoculating soybean seedlings with *Pseudomonas syringae* pv. *tomato* triggered a rapid cell death response within 12 h, which was accompanied by elevated H_2_O_2_ levels and robust expression of *PR1*, *PR5*, and cysteine protease genes. In contrast to senescence, *BiP*-overexpressing lines showed a similar increase in the expression of *GmNAC81*, a vacuolar processing enzyme (VPE) homolog gene, and SA-mediated *PR* genes, like in case of wild-type plants after *Pseudomonas spp*. infection. Moreover, H_2_O_2_ production and HR were more pronounced in BiP-enhanced tobacco leaves, and BiP suppression attenuated the HR and SA-responsive *PR1* and chitinase genes were less triggered by nonhost–pathogen interactions. This observation confirmed that BiP antagonistically modulates the SA-mediated induction of *UPR* and *PR* genes, which is coordinated with the induction of the cell death response [69]. Based on these findings, the investigation of the duration and timing of BiP accumulation, the duration of UPR, and long-term effects of SA could also be an interesting research field. In addition, activation of UPR may be regulated differently during the day and night [52] and may also be regulated by circadian rhythms like SA-regulated *PR1* expression and redox balance is [51], which has not been investigated yet. Other studies also demonstrated the role of VPE in ER stress and cell death [76], which controls tonoplast rupture, confirming the potential relationship between ER and other compartments. A lethal concentration of SA induced the expression of *SlVPE1* and the antiapoptotic Bax inhibitor-1 (*SlBI-1*) in tomato roots within three hours after exogenous 1 mM SA treatment, but in the case of sublethal treatment (0.1 mM), transcript levels of *SlVPE1* and *SlBI-1* did not change [77]. This observation may imply the potential role of SA in the coordination of ER stress and proteolysis under PCD [16]. However, BI-1 is involved in the inhibition of PCD in *Arabidopsis* by decreasing ER stress-induced ROS production or by regulating Ca^2+^ homeostasis [78,79]. Not only can the vacuolar membrane be destroyed by SA during HR and PCD, but other membrane structures can also be involved, such as membranes of chloroplasts or mitochondria [57,58]. Thus, compositional changes in the ER membrane, such as in the phospholipid content and distribution upon SA treatment, can be also important signaling events to promote ER stress [49,80]. Polyamines (PA), such as spermine (Spm), could be significant candidates for the activation of UPR. Namely, it was found that Spm induces UPR by activating the splicing of the bZIP60 transcript mediated by IRE1 [81]. It is also well-known that SA in a concentration- and time-dependent manner regulates PA metabolism in plants [82], but the potential relationship between SA and UPR under mild and chronic ER stress is not known.

Not only BiP and PDI but also CRT play a role in plant immunity [83]. SA accumulation was significantly increased in *Arabidopsis* overexpressing *CRT2*, which was associated with the activation of the transcription of *PR1,2* and *5* genes but displayed reduced resistance to virulent *Pseudomonas syringae* pv. *tomato* DC3000 [84]. Based on this observation, CRT2 can act as a self-modulator, which plays a role in the fine-tuning of the SA-dependent immunity triggered by its Ca^2+^-buffering activity, and may prevent runaway defense responses through the N-terminal domain required for chaperone activity [84]. In contrast, the role of CRT3 is associated to ethylene, because *PR1* expression did not change in CRT3a-silenced tobacco in disease resistance against the oomycete pathogen *Phytophthora infestans* [85]. These results also suggest that the physiological responses to infection are highly dependent on phytohormone interactions and SA and ET/JA levels [43]. Thus, investigation of SA together with other defense-related phytohormones in UPR could be an important future challenge. 

## 4. Concluding Remarks and Future Perspectives

Under various abiotic and biotic stresses, protein synthesis and folding in ER can be inhibited or damaged, leading to the accumulation of misfolded or unfolded proteins in the lumen of ER, thus promoting ER stress and UPR. In plants, different ER stress signaling pathways have been identified, which investigated the ER membrane-bound stress sensors IRE1 and bZIP28 or NAC TFs. Under ER stress, the IRE1-RIDD pathway was induced to cleave mRNAs attached to the ER membrane, thus preventing further protein synthesis. Activated bZIP60 TF is translocated to the nucleus and it upregulates *UPR* genes, such as various chaperones. If UPR is incapable of decreasing ER stress, autophagy and PCD can be induced. 

Based on the reviews of the existing literature, there is a link between ER stress responses and SA in plants. However, future studies are needed to reveal how SA modulates the sensing and signaling of ER stress. The time-, concentration-, species-, organ-, and cell-dependent role of SA requires more in-depth studies. The following questions have to be answered: 

What is the role of SA in the switch from life to PCD during ER stress? What is the relationship and crosstalk between ER and other organelles in this process? How is SA involved in the co-operation with other phytohormones in cell fate determination upon ER stress? What terminates UPR and inactivates IRE1? How is chaperone synthesis regulated by phytohormones?

Understanding ER stress and defense activation represents an important future challenge. A deeper knowledge of the role of phytohormones in ER stress and UPR can help to design novel strategies for ER stress and plant protection management in agricultural research.
